# Asymmetry between nasal and temporal refraction with accommodation in myopes and emmetropes

**DOI:** 10.1364/BOE.578852

**Published:** 2026-01-09

**Authors:** Shrilekha Vedhakrishnan, Charlie Börjeson, Faik Ozan Özhan, Alberto Dominguez Vicent, Abinaya Priya Venkataraman, Linda Lundström

**Affiliations:** 1 Department of Applied Physics, Royal Institute of Technology (KTH), Stockholm, Sweden; 2 Division of Eye and Vision, Department of Clinical Neuroscience, Karolinska Institute, Stockholm, Sweden

## Abstract

This study investigates relative peripheral refraction (RPR) in emmetropic and myopic eyes in the 25° nasal and temporal visual fields under far and near fixation, with control for any fluctuations in accommodation. Additional analysis of axial length and comparison with recently published eye models are also presented, constituting complementary adult data to the Stockholm Myopia Study. In the ten emmetropes, a pronounced nasal-temporal asymmetry was observed, with significantly more myopic RPR nasally and less myopic / more hyperopic RPR temporally at both accommodative states (*p* = 0.005). The nine myopes, in contrast, exhibited more symmetric peripheral profiles, with no significant nasal–temporal differences. Accommodation induced systematic shifts in both groups, producing increased relative myopia nasally and relative hyperopia temporally (*p* < 0.001). Axial length was significantly correlated with temporal hyperopic shifts during accommodation in myopes (*p* = 0.005), suggesting a structural contribution of ocular growth to peripheral optics. Comparison with eye models showed partial agreement, though experimental results revealed greater asymmetry than predicted in emmetropes and a weaker nasal–temporal distinction in myopes. Our findings indicate that variations in relative peripheral refraction over the horizontal visual field and with accommodation might be linked to ocular growth and are important for optical myopia control.

## 1. Introduction

The link between myopia and image quality on the peripheral retina continues to elude vision researchers and specialists. It has often been proposed that the human eye, just as in many animals, responds to hyperopic defocus by increasing axial length to bring the peripheral image closer to the retina [1–4], although other aspects of the peripheral image may also form ocular growth signals. In addition, eye diseases affecting peripheral, or both peripheral and foveal, vision have been associated with myopia [5,6], whereas diseases affecting only foveal vision, including maculopathy, have been linked to mild hyperopia [7,8]. Myopia has also been associated with near work. Accommodative responses in myopes are typically poorer than in emmetropes, blurring the retinal image, and extensive, prolonged near vision has been suggested to stimulate the development and progression of myopia [9–12].

High myopia is a risk factor for sight-threatening diseases, such as retinal detachment, glaucoma etc. [13–17].The most generic form of myopia develops during childhood because of exaggerated ocular growth during the emmetropization process. The eye, therefore, becomes too long compared to its refractive power and requires negative correction to see distant objects sharply. Multiple factors likely underlie the rising percentage of children worldwide who develop myopia, and several methodologies have been proposed to reduce its progression. Some of the most promising approaches use specially designed spectacles or contact lenses to manipulate image quality on the peripheral retina [18–20]. While a common explanation for their efficacy is that these lenses impose myopic (negative) relative peripheral refraction (RPR), i.e. placing the peripheral image shell in front of the retina while keeping the foveal image in focus, peripheral image quality is determined by more than defocus alone. Off-axis aberrations, e.g. oblique astigmatism and coma, together with peripheral defocus and pupil size collectively determine the peripheral modulation transfer function. Consequently, these lenses may not only shift the peripheral image shell forward but may also affect higher order aberrations that could influence ocular growth, in addition to the sign and magnitude of defocus [3,21–24]. Recent work has expanded on this view, emphasizing that modern myopia control strategies are likely to act through multiple complex visual signaling pathways [25,26].

Previous studies have compared RPR in emmetropic and myopic eyes and found that myopic eyes usually have less negative/more positive RPR in the mid periphery (20-30°) [27–31]. However, assessing RPR is methodologically challenging, and results often vary across individuals and experimental setups. Because RPR is defined as the difference in refractive error between the peripheral and the central field, any variation in the state of accommodation between the foveal and the peripheral measurement can compromise results. Differences in fixation stability, target distance, and measurement sequence can further amplify these effects. Such variability may partly explain why previous findings on the change in RPR with accommodation are inconclusive [27,28,30–35]. The relationship between myopia, near work, and peripheral refraction across field angles and refractive groups therefore remains unresolved.

The aim of the current study is therefore (1) to measure RPR at 25° off-axis in the nasal and the temporal visual fields, with and without accommodation, in emmetropic and myopic young adults using simultaneous evaluation of the central and peripheral field; (2) to summarize the methodologies and findings of previous studies and relate them to new results; and (3) to compare the experimental data to theoretical results using Zemax eye models from previous studies [36–38]. The data provided will be important in evaluating and improving the design of optical myopia control interventions. The study is part of the Stockholm Myopia Study [39] and the data provided will be important in evaluating and improving the design of optical myopia control interventions.

## 2. Methods

### 2.1. Measurements

The study was conducted according to the tenets of the Declaration of Helsinki and was approved by the Swedish Ethical Review Authority. Before the start of the experiment, each subject was provided with a detailed description of the measurement process and the purpose of the study. An informed, written consent was obtained from every subject. The inclusion criteria were best corrected visual acuity of at least 1.0 decimal (20/20), no reported ocular or binocular vision disorders, astigmatism ≤0.75 D, and spherical equivalent within ±0.5 D for the emmetropic group and from −1.5 D to −5 D for the myopic. Refraction was confirmed with an autorefractor (Wave Analyzer Medica 700 Essilor Instruments, USA) without cycloplegia. The final sample consisted of nine myopic and ten emmetropic subjects.

The RPR measurements were performed in a dual-angle open field wavefront sensor. The system has two measurement channels, each channel consisting of a Hartmann-Shack wavefront sensor, which enables data acquisition at two visual field locations at the same time (fovea and one 25° peripheral location). It records wavefronts continuously at an acquisition rate of approximately 6 Hz per channel, which means that a 10 second measurement would give 60 foveal-peripheral wavefront pairs. The system also allows for binocular viewing of fixation targets through a hot mirror, thus creating natural accommodation conditions. The main advantage of this open view system is that it minimizes instrument-induced accommodation and fixation artifacts that can arise in closed-view aberrometers. A more detailed description of the system is provided in previous publications [28,33,39].

Four measurements with three repetitions (each lasting around 10 seconds) were performed for the right eye of each subject in the following order:

• Fovea + 25° temporal visual field with relaxed accommodation (far target at 0.22 D)
• Fovea + 25° temporal visual field with accommodation (near target at 5 D)
• Fovea + 25° nasal visual field with accommodation (near target at 5 D)
• Fovea + 25° nasal visual field with relaxed accommodation (far target at 0.22 D)

**Fig. 1.**
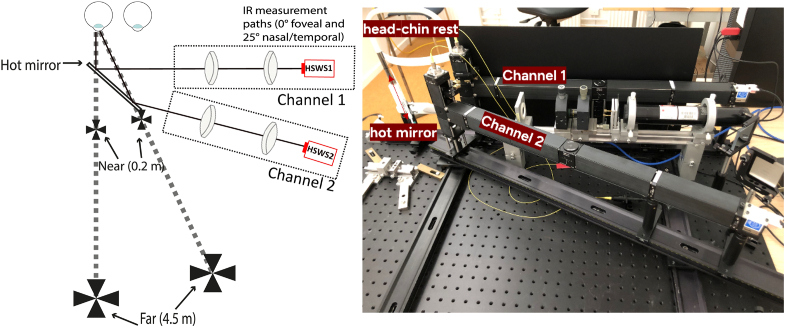
Illustrations of the measurement setup for recording two wavefronts simultaneously for the right eye - one in the fovea and one in a peripheral field (25° nasal or temporal). Left: Schematic drawing showing the hot mirror, the two channels, and the fixation targets. Fixation on the left Maltese crosses (as shown in the drawing) means that Channel 1 measured the foveal and Channel 2 the nasal visual field, whereas fixation on the right crosses means that Channel 1 measured the temporal and Channel 2 the foveal visual field. Right: Actual photograph of the setup showing the two channels, head-chin rest, and hot mirror.

During the experiment, the subjects viewed illuminated Maltese crosses (black-and-white) subtending 2° in diameter. As shown in Fig. 1, the Maltese crosses were aligned with the tested eye so when the subject accommodated only the fellow eye converged and not the measured eye. A head-chin rest and a pupil camera were used for stabilizing and aligning the subjects with the measurement axis of the system, more precise alignment was also done by monitoring the centration of the foveal and peripheral spot patterns from the wavefront sensors. The ambient light in the experimental room was dimmed to minimize distractions. The myopic group wore their habitual correction either as contact lenses or spectacles during the measurements. The subjects were given breaks whenever they needed and also in between each condition. The experiment also included biometric measurements (axial length and lens tilt), obtained using Eyestar 900 (Haag-Streit AG, Switzerland)

### 2.2. Data analysis

The raw spot pattern images from the wavefront sensors were manually reviewed, and any images with strong corneal reflections, misalignment, eyelid obstruction or poor quality were removed. As the dual angle system captures foveal and peripheral wavefronts simultaneously, any wavefront without a corresponding pair was also excluded. The images were then processed in MATLAB, where wavefronts were reconstructed using Zernike polynomials. Chromatic aberrations were compensated by converting reconstructed Zernike coefficients from measured wavelength of 830 nm to 550 nm. Both foveal and peripheral refraction were then calculated using the 2^nd^ order Zernike coefficients. The spherical equivalent values derived from each wavefront were reviewed manually to detect reconstruction errors or substantial accommodative fluctuations, with any such cases excluded from further analysis. After this sorting each condition still had at least 90 good wavefront pairs (nasal/temporal with their corresponding foveal) per measurement condition.

RPR was then determined as the difference between peripheral and foveal refraction (spherical equivalent, M):

$$\text{RPR }=\;{\text{M}}_{periperal}-{\text{M}}_{foveal}$$

For each repetition, median RPR values were obtained for nasal far, nasal near, temporal far and temporal near. RPR was then defined for each subject and condition as the average of the median RPR from the three repetitions. The difference in RPR between near and far (RPR change with accommodation) was calculated by:

$$\text{RP}{\text{R}}_{change}=\text{ RP}{\text{R}}_{near}-\text{ RP}{\text{R}}_{far}.$$

The RPR was calculated both for natural pupil size and for pupils scaled to 3 mm diameter, as all subjects had pupils larger than 3 mm. Statistical analysis was performed with SPSS software Statistics 24.0 (IBM, United States). The significance in asymmetry between the temporal and nasal visual fields was assessed with Wilcoxon signed rank test.

### 2.3. Comparison with eye models

Three recent wide-angle eye models were evaluated and compared with our experimental data: the decentered model of Atchison et al. [38], in which the lens and retina are tilted and displaced relative to the optical axis for an emmetropic and a myopic (-4 D) eye in the unaccommodated state; the emmetropic unaccommodated model of Akram et al. [37]; and the two typical eye models (-0.02 D and -2.75 D) of Hastings et al. [36] as well as their 28 emmetropic and 20 myopic individual eye models at accommodative demands matching the experimental procedure (0.22 D far, 5 D near). For convenience, they are here referred to as Atchison, Akram, and Hastings eye models, respectively. All models consist of four refracting surfaces and include a gradient index (GRIN) crystalline lens. They are optimized using the ZEMAX ray tracing program based on actual wavefront measurements and considering anatomically realistic parameters and asymmetries. The line of sight is the reference axis for Hastings and Atchison eye models, whereas the Akram eye model, although not explicitly based on it, is closely aligned with this axis. The Hastings and Atchison eye models are based on ray tracing from object space to the retina. Akram eye model used a backward ray tracing scheme from the retina to object space.

Based on the ZEMAX lens files provided by the authors, we extracted the refraction of the eye models for 0° and ±25° visual fields based on second-order Zernike aberrations at 550 nm and calculated the nasal and temporal RPR. Since the Akram eye model provides configurations only for specific angles (from −40° to 40° in 10° increments), we generated configurations for ±25° visual fields using geometric construction. In contrast, the Hastings models are based on ray tracing from object space to the retina, allowing us to directly specify the object field angle for the three angles of interest. We used the ZEMAX Programming Language (ZPL) to extract refraction values from the 2^nd^ order Zernike coefficients at these angles for the Akram and the Hastings models. For the Atchison eye model, we directly obtained the RPR values from the authors of the paper.

## 3. Results

### 3.1. Participants

Table 1 shows data on age, refraction, and ocular biometry for the individual subjects as well as the average of the two refractive groups.

**Table 1.** Individual subject data of the right eye. Standard deviations for mean values are given within parenthesis. SNO = subject number, Age, M = spherical equivalent, Cyl - Astigmatism, AL = axial length, LTA = lens tilt axial angle (angle between lens axis and corneal vertex normal), LTR = lens tilt rotational angle (direction of the tilt), LDX = lens decentration x-axis, LDY = lens decentration y-axis, HC = habitual correction with either contact lenses (CL) or spectacles (S), E/M = classified as emmetropic/myopic

SNO	Age (years)	M (D)	Cyl (D)	AL (mm)	LTA (°)	LTR (°)	LDX (mm)	LDY (mm)	HC	E/M
1	22	-3.25	-0.75	25.22	3.63	184.55	-0.05	+0.08	CL	M
2	26	-4.5	-	24.84	3.29	215.68	-0.02	+0.14	CL	M
3	25	-2	-0.25	23.82	5.02	185.56	-0.09	+0.07	CL	M
4	22	-1.5	-0.25	24.66	2.66	206.9	0	+0.09	S	M
5	31	-2.25	-	24.95	2.92	214.61	+0.01	+0.01	S	M
6	24	-2	-	24.83	3.05	203.03	-0.01	+0.10	CL	M
7	20	-1.5	-	23.84	3.43	186.37	+0.01	+0.12	S	M
8	26	-2	-0.25	25.17	2.80	206.64	+0.10	+0.11	CL	M
9	37	-3	-0.5	24.41	4.24	166.65	-0.08	+0.20	CL	M

**Mean Myopes**	**25.4 (±2.58)**	**-2.45 (±0.45)**	**-0.25 (±0.13)**	**24.6 (±0.2)**	**3.44 (±0.38)**	**196.66 (±8.30)**	**-0.01 (±0.02)**	**0.10 (±0.02)**		

10	27	-	-0.75	22.3	4.51	195.55	-0.10	+0.04	-	E
11	24	-	-	23.01	2.89	197.67	-0.06	+0.12	-	E
12	31	+0.25	-	23.51	3.42	193.28	+0.01	+0.11	-	E
13	28	+0.5	-	23.69	2.65	167.8	0	+0.13	-	E
14	34	+0.5	-	23.37	4.40	181.71	-0.05	+0.17	-	E
15	22	+0.25	-	22.42	3.48	191.34	-0.11	+0.09	-	E
16	21	-	-	22.83	4.18	180.01	0	+0.12	-	E
17	28	-	-	23.66	4.86	194.22	-0.05	-0.02	-	E
18	24	+0.5	-	23.95	4.20	209.51	-0.15	+0.19	-	E
19	28	-	-	24.54	3.95	165.26	+0.06	+0.21	-	E

**Mean Emmetropes **	**26.7 (±2.01)**	**+0.2 (±0.11)**	**-0.07 (±0.11)**	**23.32 (±0.34)**	**3.85 (±0.38)**	**187.13 (±5.94)**	**-0.04 (±0.02)**	**0.11 (±0.03)**		

### 3.2. Median relative peripheral refraction for nasal and temporal visual fields

**Fig. 2.**
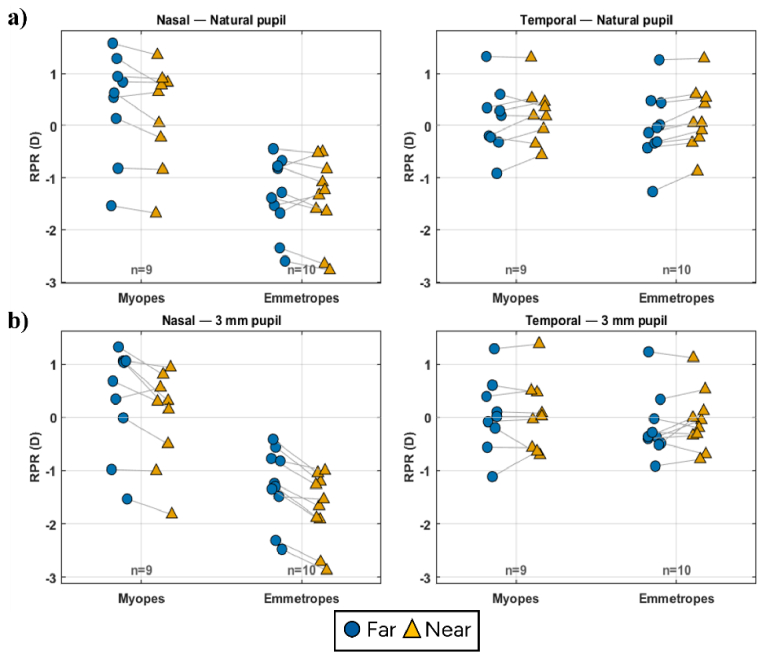
Median relative peripheral refraction for both nasal (left) and temporal (right) visual fields plotted for all subjects myopes (9) and emmetropes (10). The median RPR is shown for both far (blue round markers) and near (yellow triangle markers). These results are shown with natural pupils (a) and for 3 mm cropped pupils (b). Connecting lines are included to show far and near of the same subject.

Figure 2(a) shows the median RPR, evaluated for natural pupil size, for both nasal and temporal visual fields for each individual emmetrope and myope separately. For far fixation, temporal RPR values ranged from –1.2 to +1.3 D, while nasal RPR values ranged from –2.6 to +1.5 D. For near fixation, the corresponding ranges were –0.9 to +1.3 D temporally and –2.7 to +1.3 D nasally, when data from both groups were combined. The average RPR in myopes was 0.4 D/0.2 D for far and near in the nasal field and 0.12 D/0.2 D for far and near in the temporal field. The emmetropes had on average a negative RPR of -1.4 D/-1.4 D for far and near in the nasal field and -0.0D/0.1D for far and near in the temporal field. Hence, there was a significant asymmetry in average RPR for the emmetropes; in the nasal it was myopic whereas it became hyperopic or less myopic in the temporal visual field in both accommodation levels (-1.3 D, p = 0.005 for far, -1.5 D, p = 0.005 for near, Wilcoxon signed ranks test). On the contrary, there was no significant asymmetry in RPR between the nasal and temporal visual fields for myopes in either accommodation levels (+0.2 D for far, -0.02 D for near). There was a significant difference in RPR between the emmetropic and myopic groups in the nasal field for both far (p = 0.011, Wilcoxon signed ranks test) and near (p = 0.011, Wilcoxon signed ranks test). Also, when excluding the effect of pupil size by cropping to a uniform pupil size of 3 mm, the same trends were found across both groups (Fig. 2(b)).

### 3.3. Relative peripheral refraction change with accommodation

**Fig. 3.**
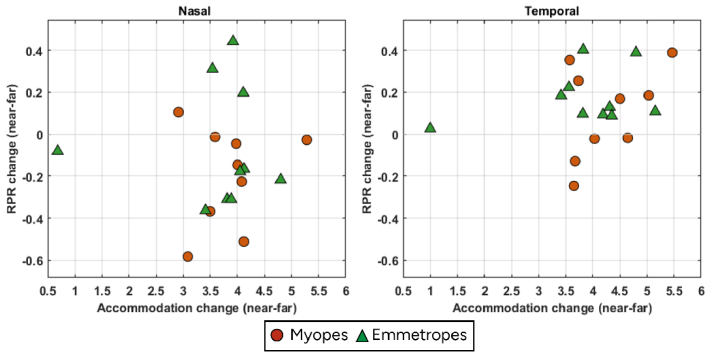
Relative peripheral refraction changes with accommodation from far to near for both nasal (left) and temporal (right) visual fields in myopes (red round markers) and emmetropes (green triangle markers).

Figure 3 shows the RPR change with accommodation from far to near for both nasal and temporal visual fields for the emmetropic and myopic group. This RPR change was significantly different between myopes and emmetropes in the nasal (p = 0.008, Wilcoxon signed rank test) as well as in the temporal visual field (p = 0.037, Wilcoxon signed rank test). Overall, within both groups there was also a significant difference between the temporal and nasal visual fields in how RPR changed with accommodation (p < 0.001, Wilcoxon signed rank test in both myopes and emmetropes). On average, across both groups, accommodation caused more relative peripheral myopia/less relative peripheral hyperopia in the nasal field (myopes: -0.20 D; emmetropes: -0.06 D), while in the temporal visual field it caused more relative peripheral hyperopia/less relative peripheral myopia (myopes: 0.10 D; emmetropes: 0.17 D). It can be noted that one of the emmetropic subjects showed very minimal accommodation which could be due to fatigue or reduced effort during the task.

### 3.4. Relative peripheral refraction change with axial length

**Fig. 4.**
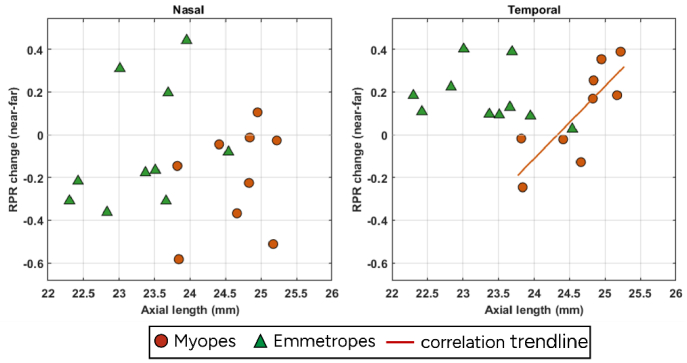
Relative peripheral refraction changes with axial length for both nasal (left) and temporal (right) visual fields in myopes (red round markers) and emmetropes (green triangle markers). The red trendline shown in temporal myopes indicates the positive correlation with axial length.

Figure 4 shows the RPR change with accommodation as a function of axial length for both nasal and temporal visual fields in myopes and emmetropes. There was a significant correlation between temporal RPR change in myopes with axial length (p = 0.005, Spearman’s rho correlation). This correlation was positive (R^2^ = 0.17), indicating that a longer axial length was associated with a more hyperopic shift in temporal RPR of myopes during accommodation. Overall, myopes had longer axial length than emmetropes, as expected. We did not find any correlation between lens tilt and RPR change.

### 3.5. Predictions from eye models

**Fig. 5.**
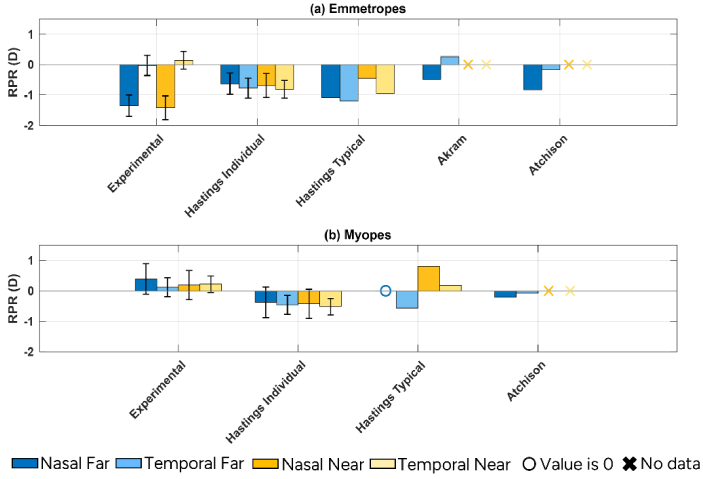
Comparative evaluation of RPR across Zemax computed eye models from three publications and current experimental data across nasal (dark bars) and temporal (light bars) under varying accommodative demands – far (blue) and near (yellow). These studies include both emmetropic (a) and myopic (b) subjects. The results shown here are for 25° nasal and temporal visual fields. If there was no data, then it is indicated by X and if the value is 0 then it is indicated with 0 marker in the corresponding colors.

The RPR of the eye models for the 25° nasal and temporal visual fields are presented together with the average experimental data in Fig. 5. For an emmetropic eye without accommodation, results from the Atchison model mostly agree with the experimental findings. The model also reproduces the difference in nasal RPR between emmetropes and myopes without accommodation. The temporal RPR value of the emmetropic Akram eye model is more hyperopic than the nasal RPR, which also aligns well with the experimental data. However, the values are more hyperopic than the experimental averages. The nasal RPR value of Hastings typical emmetropic eye model closely matches experimental data without accommodation. With accommodation, no correlation is observed between experimental data and the Hastings eye models.

For the myopic eye in the unaccommodated state, the temporal RPR value of Hastings typical eye model is more myopic than the nasal RPR, consistent with experiments. However, both Hastings typical and the average of Hastings individual models predict more myopic values compared to experimental data. With accommodation, temporal RPR of Hastings typical model is consistent with the experimental results. Also, the hyperopic shift with accommodation in the temporal, but not in the nasal, RPR of the model is consistent with experimental data.

## 4. Discussion

This study investigates the relative peripheral refraction over the horizontal visual field (mid periphery 25°) with and without accommodation in myopes and emmetropes. We found that emmetropes showed significant nasal-temporal asymmetry, with more myopic RPR nasally and less myopic/hyperopic RPR temporally for both far and near fixation, whereas myopes showed no significant asymmetry. With accommodation, the RPR change was significantly different between myopes and emmetropes in both visual fields, with myopes showing the largest negative shift in the nasal field and emmetropes the largest positive shift in the temporal. Also, axial length analysis revealed a significant positive correlation between temporal RPR change and axial length in myopes.

### 4.1. Literature review

**Fig. 6.**
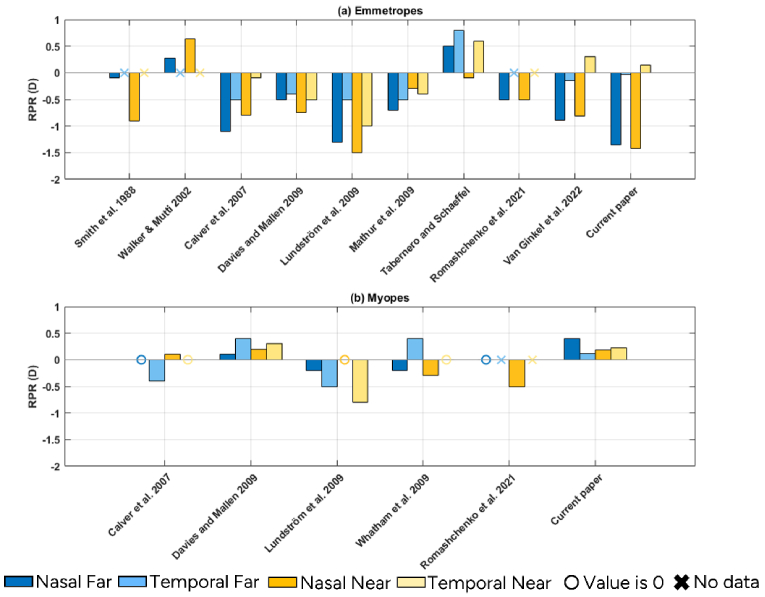
Comparative evaluation of RPR across various studies, highlighting the differences in measurements across nasal (dark bars) and temporal (light bars) visual field under varying accommodative demands – far (blue) and near (yellow). These studies include both emmetropic (a) and myopic (b) subjects. The results shown here are for 20-30° nasal and temporal visual fields. If there was no data, then it is indicated by X and if the value is 0 then it is indicated with 0 marker in the corresponding colors.

An analysis of peripheral refraction data from several published studies [27, 28, 30–32,34, 35, 40–42] was performed to compare with our current study. This comparative evaluation is shown in Fig. 6 for both nasal and temporal RPR at visual angle between 20-30°. Except for Romashchenko et al. [28] all the other studies have followed methodologies different from ours for measuring RPR: most of them have used Shin-Nippon autorefractor with monocular or binocular viewing of the targets and have performed time-separate foveal and peripheral measurements. This makes our study novel as we were able to measure simultaneously at the fovea and nasal/temporal periphery allowing us to calculate RPR accurately in a natural binocular setting for two levels of accommodation. As shown in the plot, many of the studies have included emmetropes and only a few studies included myopic subjects. In the current study, RPR values for myopic eyes were consistently more hyperopic than those for emmetropic eyes, in agreement with previous studies [28, 30–32]. In emmetropes, our results are also consistent with earlier research showing more negative RPR nasally than temporally, though the magnitude may vary due to differences in participant age, refractive error, or measurement methodology [30–32,35,43,44]. Furthermore, Smith et al, Davies et al., Lundström et al, and Tabernero et al. all found more negative RPR with accommodation in the nasal visual field of emmetropes, which was also seen in our study.

### 4.2. Asymmetry in RPR over the horizontal field

The strong asymmetry between nasal and temporal RPR found for the emmetropic subjects in this and previous studies is likely primarily due to the increase in off-axis astigmatism with eccentricity, as the tangential image plane shifts in the myopic direction with the field angle measured from the eye’s optical axis, in combination with the temporal displacement of the fovea in relation to the optical axis. Since the off-axis angles given in this study, as well as in earlier studies, refer to the line of sight, this results in measured eccentricities that are not optically symmetric. Finding the true optical axis of the eye is challenging, and we therefore do not know the actual off-set between the line of sight and the optical axis in our subjects. However, if we assume that this off-set is similar to angle alpha, i.e. that the line of sight lies approximately 3-5° nasally of the optical axis in the visual field [45], this means that our 25° measurements correspond to 28-30° nasally and 20-22° temporally relative to the optical axis. This inherent asymmetry in measurement geometry is therefore expected to result in more myopia in the nasal than in the temporal visual field [45–48]. Additionally, physiological crystalline lens tilt or decentration can alter peripheral optical quality differently across the field. These tilts have been shown to introduce asymmetries in defocus and astigmatism, which could further contribute to the nasal-temporal differences [48–50]. However, in our current study we did not find any specific correlation between lens tilt and RPR change.

The absence of nasal–temporal asymmetry in the myopic group observed in our study is consistent with previous reports. Variations in peripheral optics between myopic and emmetropic eyes are influenced by both anterior and posterior ocular structures. Posteriorly, myopic eyes typically exhibit a more prolate retinal contour, which is associated with reduced or absent nasal–temporal asymmetry in peripheral refraction [51]. Anteriorly, myopic eyes have been shown to display altered corneal geometry, characterized by reduced nasal–temporal asymmetry relative to emmetropic eyes [52]. Supporting this, a study [53] examining changes following orthokeratology treatment reported that relative peripheral hyperopia in myopic eyes was converted to relative peripheral myopia, and the nasal–temporal asymmetry changed significantly in association with the baseline refractive state. The diminished optical asymmetry observed in myopic eyes compared with emmetropic eyes may modify the ocular growth feedback mechanism, thereby influencing the onset and progression of myopia [54].

In our study, measurements were obtained with myopic subjects wearing their habitual correction, 6 out of the 9 subjects used soft contact lenses. Atchinson et al. [29] showed that spectacle lenses can generate off-axis aberrations and induce peripheral hyperopic defocus, especially in the temporal visual field. In contrast, soft contact lenses tend to maintain the inherent peripheral refraction profiles, avoiding many of the oblique incidence effects typical of spectacle lenses. Vera-Diaz et al. [55] also demonstrated that differences in correction modality significantly change the measured peripheral refraction profiles. Importantly, while such corrections can shift the overall level of relative peripheral refraction, they should not introduce strong nasal-temporal asymmetries if properly aligned on the eye. Therefore, the lack of asymmetry that we observed in myopic subjects is unlikely to be an artifact of the correction modality and most likely reflects underlying ocular optics. Also, measurement under habitual correction provides important real-world context and captures optical effects relevant to everyday vision.

### 4.3. Comparison to eye models

RPR values from the eye models we studied show a similar trend in the nasal visual field for emmetropes, but one and the same model could not replicate all characteristics of the experimental data. Modelling the peripheral optics of the human eye is challenging for several reasons. Firstly, peripheral image quality is characterized by more optical errors than defocus, such as astigmatism, spherical aberration and coma, which make the location of the circle of least confusion ill-defined and more dependent on pupil size and shape.

The second major issue with designing model eyes is the large individual variations in biometry and peripheral aberrations, which make it difficult to develop a single model that represents all subjects. For example, as shown in Fig. 5, RPR values from the Hastings myopic individual eye models differ from those of their typical myopic model, especially with accommodation. This suggests that it is more challenging to develop a representative model for myopes compared to emmetropes.

Thirdly, eye models are of course generalizations and cannot have as many degrees of freedom as the natural eye has. Specifically, the Atchison model does not account for toroidal cornea. The Akram model shows limitations in estimating the trend of spherical aberration in the periphery, which may be related to constraints of its GRIN profile. If the spherical aberration term was to be included in the defocus calculations, the results of this model would show larger deviations from experimental data. The Hastings models have a fixed pupil position coinciding with the reference axis. However, in practice, the pupil center shifts with accommodation varies among subjects. In addition, Hastings typical model suggests that horizontal coma decreases from the nasal to the temporal visual field, which differs from some previous experimental findings. This might be related to the unusually large vertical lens tilt in the model compared to anatomical data. We should therefore be careful with relying on generic eye models for explaining peripheral refraction, especially for myopic eyes.

### 4.4. Importance for myopia development

Our findings on RPR in adults raise the question of how similar mechanisms may operate in children, where emmetropization is still active and peripheral optical cues may be especially relevant. Hence, we are currently performing the Stockholm Myopia study using the same research equipment [39], where we measure RPR at ±25° horizontal field for two levels of accommodation (0.22 D and 5 D) in 31 children aged 6 to 11 years. The mean cycloplegic SER of the children was +0.61 D (±1.14 D). Those results showed that RPR was larger and more negative in the nasal visual field than in the temporal and that this difference increased with accommodation. Many of the children had asymmetric RPR especially with increased accommodation. These results agree with the trends found in the emmetropic adults in the current study. In a recent publication, Vera-Diaz et al. [56] investigated longitudinal changes in peripheral optical quality (horizontal meridian ±30°) in young children at high or low risk of developing myopia, as well as in a small subgroup who had recently developed myopia. Children at high risk for myopia and those already myopic showed distinct peripheral optical profiles (less positive spherical aberration, more negative peripheral defocus and lower higher order aberrations) in the central and near peripheral retina (±20°, temporal retina) before the axial elongation occurred. Another recent study also supports that peripheral image quality can affect ocular growth in children, showing that multifocal IOLs may provide a myopia control benefit in pediatric cataract patients by inducing peripheral myopic defocus [57].

However, there are also findings that RPR does not precede or accelerate myopia, suggesting that the change in RPR is instead a consequence of the ocular growth [58]. Previous studies [43,59–67] have investigated peripheral refraction and its association with retinal shape in myopia development. Retinal shape can be estimated using imaging methods such as MRI or OCT but is more commonly inferred from peripheral refraction patterns. Steep prolate retinal shape with relative peripheral hyperopia was found in myopes, and relatively flatter retinal shape (oblate) with peripheral myopia were reported in emmetropic eyes. Much like peripheral refraction, retinal shape is also reported to vary with the meridian and eccentricity.

It should be noted that the measurements in our current study were conducted without cycloplegia as Lu et al. [68] found that peripheral refraction exhibited a significant hyperopic shift after cycloplegia that increased with eccentricity, i.e. this shift in amplitude was larger for peripheral refraction than for central refraction. Furthermore, the hyperopic shift in peripheral refraction was larger in the high myopia group than in the low-to-moderate myopia group. Therefore, it is imperative to consider the influence of accommodation on RPR under different working distances during daily activities to be able to investigate whether RPR is linked to myopia development and myopia control.

## 5. Conclusion

We have measured RPR for two levels of accommodation (0.22 D and 5 D) in our unique research instrument, which allows for simultaneous measurements in foveal and peripheral angles. Emmetropes exhibited significant nasal-temporal asymmetry, whereas myopic eyes demonstrated more symmetric nasal–temporal RPR characteristics. Accommodation induced opposing changes across the visual field, further increasing the asymmetry with increased negative shifts in nasal visual field and positive shifts in the temporal visual field. Together, these results suggest that asymmetries in peripheral refraction and their accommodative modulation may be optical cues for eye growth regulation. In conclusion, our study underscores the importance of considering the difference between nasal and temporal visual field of an adult eye, both with and without accommodation. These insights can improve the development of more effective, individualized strategies for managing myopia progression.

## Data Availability

Data underlying the results presented in this paper are not publicly available at this time but may be obtained from the authors upon reasonable request.
